# High-fat diet intake induces depressive-like behavior in ovariectomized rats

**DOI:** 10.1038/s41598-019-47152-1

**Published:** 2019-07-22

**Authors:** Valter T. Boldarine, Amanda P. Pedroso, Nelson I. P. Neto, Ana P. S. Dornellas, Cláudia M. O. Nascimento, Lila M. Oyama, Eliane B. Ribeiro

**Affiliations:** Department of Physiology, Universidade Federal de São Paulo UNIFESP, São Paulo, SP Brazil

**Keywords:** Obesity, Quality of life

## Abstract

This study tested the effects of ovariectomy, allied or not to high-fat feeding and estradiol replacement, on hormonal, metabolic and behavioral parameters, to explore the connection of obesity and depression after menopause. Wistar rats were either ovariectomized or sham-operated and fed with either standard chow or lard-enriched diet for twelve weeks. Sub-groups of ovariectomized rats received estradiol replacement. Depressive-like behaviors were assessed by the forced swim test and locomotor activity was assessed by the elevated plus maze test. Ovariectomy alone increased body weight gain and feed efficiency and induced hyperleptinemia and glucose intolerance while it increased caloric intake and body adiposity only marginally. High-fat intake alone induced obesity and, in combination with ovariectomy, accentuated the ovariectomy-induced alterations. Estradiol replacement attenuated the hormonal alterations only in chow-fed rats. Ovariectomy combined with high-fat intake induced depressive-like behaviors, which were marginally attenuated by estradiol. Depressive-like behaviors were associated with metabolic and body composition parameters and with estrogen status. The data indicate that the vulnerability to develop depression after menopause is influenced by high-fat intake. It is suggested that weight management is a crucial issue in postmenopausal women, probably having a beneficial role in preventing the appearance of mental health problems.

## Introduction

Characterized by the loss of ovarian function, menopause is a period in women´s life in which the risk of developing central obesity is higher than in any other^1–3^. Obesity brings a series of comorbidities, such as type 2 diabetes, heart diseases, and psychopathologies including mild to moderate depression^4,5^. Both epidemiological and experimental data seem to have established the existence of a bidirectional association between obesity and depression^6,7^.

Estrogen (mostly in the form of estradiol) participates in the regulation of energy homeostasis, having been shown to affect, among other mechanisms, fat accumulation and distribution, leptin sensitivity and glucose tolerance^8–10^. Moreover, acting at central nervous system sites, estradiol has been suggested to attenuate the mood and body weight alterations caused by ovariectomy in rats^11^. These considerations indicate the relevance of a better understanding of the obesity/depression association after menopause.

We have previously demonstrated that the intake of a high-fat diet by ovarictomized rats, while potentiating the ovariectomy-induced body adiposity gain and metabolic alterations, failed to affect depressive-like behaviors consistently^12^. Among middle-aged women, a group that frequently includes a high participation of post-menopausal individuals, a positive relation between depressive symptoms and obesity markers has been shown^13–15^.

The existence of a direct connection between menopause and depression itself still lacks ascertainment, since studies in both rodents and human subjects have yielded conflicting results. In rats and mice, ovariectomy has been shown to either induce depressive-like behaviors^11,16–18^, or to fail to do so^12,19–21^. In humans, the menopausal transition has been associated with high risk to develop depression^22^ while either a direct or an inverse relation between the menopausal stages and depression has been described in middle-aged women^23,24^.

All the above data indicate that menopause, obesity, and depression connect in a complex and still unascertained manner. The elucidation of these aspects are of great importance to both public health and therapeutic issues. Hence, the present study evaluated metabolic, hormonal and behavioral parameters in an ovariectomized rat model, allied or not to high-fat feeding and estradiol replacement. The main purposes of these experiments were to address the questions of whether ovarian failure associates with depression and to what extent obesity is relevant to this connection.

## Results

### High-fat intake accentuates the body weight gain and adiposity changes induced by ovariectomy

**Figure 1:**
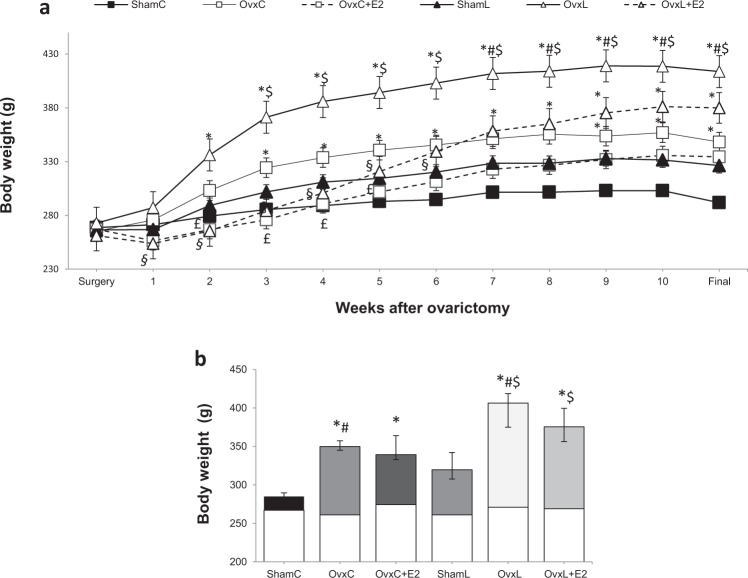
Weekly evolution of body weight (**a**) and 12-weeks cumulative body weight gain (**b**). The blank bars in Figure b represent the initial body weight. Groups were fed with either control diet - ShamC (n = 18), OvxC (n = 18), OvxC + E2 (n = 18) or high-lard diet - ShamL (n = 17), OvxL (n = 18), OvxL + E2 (n = 17), for twelve weeks. Data are expressed as medians and interquartile range (Q1–Q3) for both figures. *p < 0.05 *vs*. ShamC; ^#^p < 0.05 OvxC *vs*. OvxL; ^$^p < 0.05 *vs*. ShamL; ^£^p < 0.05 *vs*. OvxC + E2; ^§^p < 0.05 *vs*. OvxL + E2.

Figure 1a depicts the weekly evolution of body weight and Fig. 1b shows the cumulative body weight gain throughout the treatment. The animals presented similar body weight in the beginning of the treatment. Body weight gain differed significantly among the groups [H (5) = 73.21, p = 0.001]. All groups gained more weight than the ShamC group, although not significantly so in the ShamL group (157% higher, p = 0.252). Ovariectomy caused a significant increase in body weight gain, regardless the diet. Figure 1a shows that estradiol replacement reduced body weight gain during the first 5 weeks of treatment in the control-fed groups (OvxC *vs*. OvxC + E2) and during the first 6 weeks in the lard-fed groups (OvxL *vs*. OvxL + E2). However, by the end of the treatment, this effect was no longer significant.

**Figure 2:**
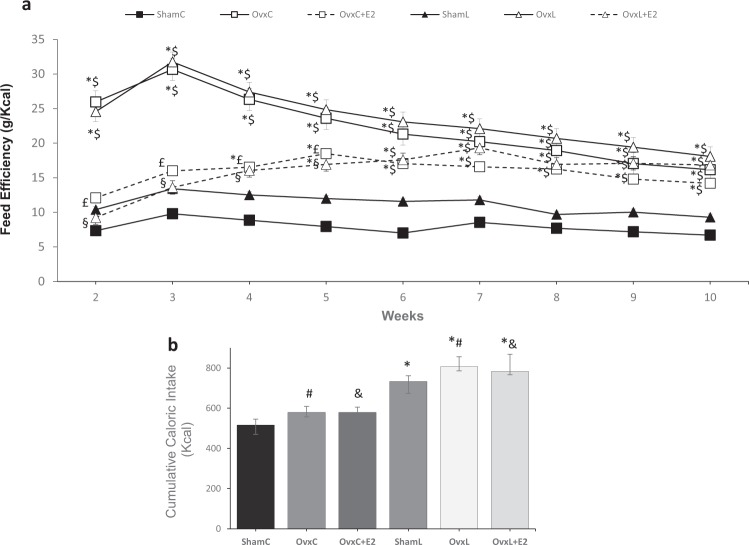
Feed efficiency (**a**) and cumulative caloric intake (**b**). Groups were fed with either control diet - ShamC (n = 18), OvxC (n = 18), OvxC + E2 (n = 18) or high-lard diet - ShamL (n = 17), OvxL (n = 18), OvxL + E2 (n = 17), for twelve weeks. Data are expressed as medians and interquartile range (Q1-Q3) for both figures. *p < 0.05 *vs*. ShamC; ^#^p < 0.05 OvxC *vs*. OvxL; ^$^p < 0.05 *vs*. ShamL; &p < 0.05 OvxC + E2 *vs*. OvxL + E2; ^£^p < 0.05 *vs*. OvxC + E2; ^§^p < 0.05 *vs*. OvxL + E2.

The feed efficiency was significantly different among the groups [H (5) = 69.21, p < 0.001] (Fig. 2a). Ovariectomy induced significantly higher feed efficiencies than those of the Sham groups throughout the treatment, regardless of the diet. Estradiol replacement was effective in reducing feed efficiency up to the 5^th^ week whereas in the remaining weeks this effect was lost, partially due to a non-significant but continuous decrease in the Ovx groups values.

The comparison of the cumulative caloric intake of the six groups [H (5) = 86.84, p < 0.001] is represented in Fig. 2b. It demonstrates that ovariectomy did not affect caloric intake significantly, independently of the diet consumed. In contrast, the intake of the high-fat diet *per se* increased caloric intake and estradiol replacement failed to alter this parameter.

Table 1 shows that the uterus weight differed among the groups [H (5) = 84.98, p < 0.001], and that ovariectomy caused a significant uterus hypotrophy. Estradiol replacement, although causing a small increase in uterus weight, did not eliminate the significant difference of the Ovx groups in comparison to the Sham groups, regardless the diet.

**Table 1:** Uterus weight, sum of fat depots and serum parameters of the groups ShamC, OvxC, OvxC + E2, ShamL, OvxL and OvxL + E2.

	ShamC	OvxC	OvxC + E2	ShamL	OvxL	OvxL + E2
Uterus (g)	0.57 (0.44–0.72) (n = 18)	0.11 (0.07–0.13)^*^ (n = 18)	0.15 (0.14–0.18)* (n = 18)	0.65 (0.47–0.69) (n = 17)	0.09 (0.06–0.09)^*$^ (n = 18)	0.15 (0.13–0.18)*^$^ (n = 17)
Sum of fat depots (g/100 g)	3.17 (2.92–3.53) (n = 18)	5.69 (5.26–6.04)^#^ (n = 18)	4.73 (4.17–5.64)^&^ (n = 18)	6.37 (5.95–7.14)* (n = 17)	7.99 (7.32–8.27)^*#^ (n = 18)	7.01 (6.76–7.65)^*&^ (n = 17)
Glucose (mg/dL)	89.1 ± 2.01 (n = 18)	95.8 ± 4.3^#^ (n = 18)	101.4 ± 4.1^&^ (n = 18)	108.1 ± 3.9^*^ (n = 17)	112.2 ± 4.3^*#^ (n = 18)	122.2 ± 4.7^*&^ (n = 17)
Insulin (ng/mL)	0.6 (0.4–0.7) (n = 10)	2.1 (1.6–2.2)^*^ (n = 10)	1.2 (1.1–1.4) (n = 10)	1.4(1.1–1.9) (n = 10)	2.1 (1.4–2.9)^*^ (n = 10)	2.2 (1.1–3.1)^*^ (n = 10)
HOMA-IR	3.8 (2.4–4.5) (n = 10)	12.1 (9.2–15–4)^*^ (n = 10)	7.9 (6.3–8.8) (n = 10)	9.6 (7.7–12.05) (n = 10)	14.5 (10.1–20.3)^*^ (n = 10)	19.3 (7.7–24.9)^*^ (n = 10)
HOMA- β	0.19 (0.15–0.26) (n = 10)	0.42 (0.37–0.49)^*^ (n = 10)	0.24 (0.22–0.34) (n = 10)	0.29 (0.17–0.36) (n = 10)	0.40 (0.21–57)(n = 10)*	0.33 (0.21–0.43) (n = 10)
Leptin (ng/mL)	2.1 (1.85–2.44) (n = 13)	8.6 (6.1–10.1)^*^ (n = 13)	6.9 (4.5–9.4) (n = 13)	5.8 (5.2–9.1) (n = 13)	12.1 (9.9–13.4)^*$^ (n = 13)	14.1 (10.9-0.6)^*$^ (n = 13)
Adiponectin (µg/mL)	5.06 (4.2–7.2) (n = 13)	7.39 (6.23–11.22) (n = 13)	5.57 (5.14–8.22) (n = 13)	5.92 (5.27–6.99) (n = 13)	6.97 (5.97–8.34) (n = 13)	5.02 (4.59–9.01) (n = 13)
Leptin/Adiponectin Ratio	0.34 (0.2–0.5) (n-13)	0.94 (0.6–1.6)^*#^ (n = 13)	0.79 (0.59–1.5)^&^ (n = 13)	0.87 (0.5–1.1) (n = 13)	1.85 (1.5–2.7)^*#$^ (n = 13)	2.43 (0.9–3.3)^*&$^ (n = 13)
Triglicerydes (mg/dL)	101.4 (93.6–106.7) (n = 18)	116.6 (104.1–26.6) (n = 18)	126.1 (97.4–131.9) (n = 18)	89.7 (81.8–115.3) (n = 17)	109.4 (88.1–117.3) (n = 18)	101 (93.1–119.8) (n = 17)
Total cholesterol (mg/dL)	106.8 (91.5–20.9) (n = 18)	113.1 (104.9–39.5) (n = 18)	120.8 (115.7–128.1) (n = 18)	103.2 (92.5–109.9) (n = 17)	98.6 (93.0–114.3) (n = 18)	112.2 (91.7–123.1) (n = 17)
HDL cholesterol (mg/dL)	39.3 (36.7–47.1) (n = 18)	34.2 (31.4–38.7) (n = 18)	38.5 (30.1–47.3) (n = 18)	35.8 (32.5–37.9) (n = 18)	32.4 (28.8–38–4) (n = 18)	38.6 (29.4–42.4) (n = 18)

Data presented as mean ± SEM for variables with normal distribution and medians- interquartile range (Q1–Q3) for variables not normally distributed.

*p < 0.05 *vs*. ShamC; ^#^p < 0.05 OvxC *vs*. OvxL; ^&^p < 0.05 OvxC + E2 vs. OvxL + E2; ^$^p < 0.05 *vs*. ShamL.

The sum of fat depots also showed to be significantly different among the groups [H (5) = 66.01, p < 0.001] in a diet-dependent manner, with the high-fat groups presenting increased fat depots compared to the respective control groups. Ovariectomy itself produced non-significant increases in fat depots in both diet groups. The intake of the high-fat diet by the Ovx rats further increased fat depots weight. Estradiol supplementation tended to decrease fat depots but the effect was not significant.

### High-fat intake impairs the ability of estradiol replacement to attenuate the deleterious effects of ovariectomy on glucose homeostasis

Also highlighted by Table 1 are the serum parameters results. Glucose levels were different among the groups (F_[5,98] = _34.12, p < 0.001), being increased in the animals that received the high-fat diet.

Significant differences in insulin levels [H (5) = 20.38, p = 0.001], HOMA-IR [H (5) = 22.43, p = 0.004] and HOMA-β [H (5) = 12.48, p = 0.028] were also present among the groups. The results showed that insulin levels and HOMA -IR were affected by ovariectomy, being higher in the OvxC and OvxL groups than in the ShamC group. On the other hand, HOMA-β increased only in the OvxC group compared to the ShamC group. Estradiol replacement was able to normalize insulin levels only on the group fed with control diet.

Leptin levels varied significantly among the groups [H (5) = 43.99, p < 0.001], with increases in the Ovx groups on both diets, compared to the respective Sham groups, demonstrating an effect of ovariectomy. Estradiol replacement was able to normalize leptin levels only in the group fed with the control diet. Leptin/Adiponectin ratio also varied among the groups [H (5) = 49.69, p < 0.001]. Adiponectin, triglycerides, and total and HDL cholesterol levels did not differ significantly among the groups.

### High-fat intake induces depressive-like behaviors in ovariectomized rats

**Figure 3:**
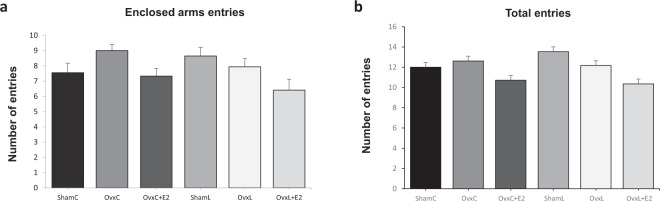
Elevated plus-maze test: Number of enclosed arms entries (**a**) and number of total entries (enclosed and opened arms). (**b**) Groups were fed with control diet-ShamC (n = 18), OvxC (n = 18), OvxC + E2 (n = 18), and groups fed with high-lard diet-ShamL (n = 17), OvxL (n = 18),OvxL + E2 (n = 17). Data are expressed as means ± SEM.

The results of the EPM test showed that both the number of entries in the enclosed arms and the total number of entries were similar among the groups (Fig. 3a,b), indicating that the treatments did not affect locomotor activity.

**Figure 4:**
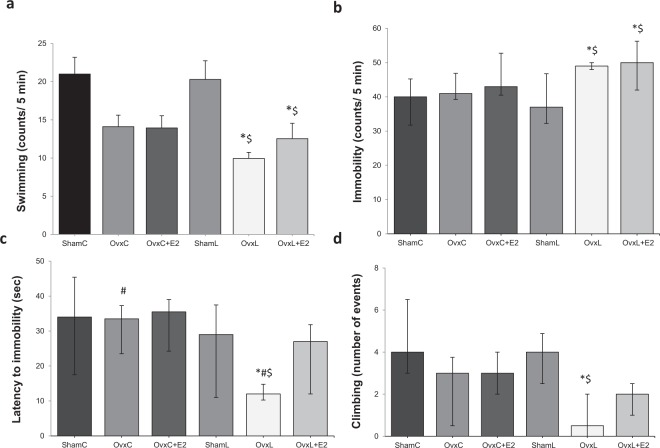
Modified forced swim test: Swimming frequency (**a**), Immobility frequency (**b**), latency to immobility (**c**) and climbing events (**d**) in groups fed with control diet-ShamC (n = 18), OvxC (n = 18), OvxC + E2 (n = 18), and groups fed with high-lard diet-ShamL (n = 17), OvxL (n = 18),OvxL + E2 (n = 17). Data are expressed as means ± SEM for swimming frequency and as medians and interquartile range (Q1-Q3) for the other parameters. *p < 0.05 *vs*. ShamC; ^#^p < 0.05 OvxC *vs*. OvxL; ^$^p < 0.05 *vs*. ShamL.

According to the FST test, swimming frequency differed significantly among the groups (F_[5,98]_ = 11.16, p = 0.001). Figure 4a shows a decrease in the swimming frequency of both OvxL and OvxL + E2 groups compared to both Sham groups.

The immobility frequency (Fig. 4b) also differed among the groups [H (5) = 27.89, p < 0.001], increasing in OvxL and OvxL + E2 groups compared to both Sham groups.

The latency to immobility showed significant differences among the groups [H (5) = 22.89, p = 0.002], with the OvxL group showing the lowest latency. Estradiol replacement normalized this parameter (Fig. 4c).

The number of climbing events differed among the groups [H (5) = 26.82, p = 0.001], with significantly fewer events showed by the OvxL group when compared to the ShamC and ShamL groups. Estradiol replacement normalized this parameter (Fig. 4d).

### Obesity and lack of estradiol associate with behavioral parameters

The following parameters were included in the correlation analysis: body weight gain, feed efficiency, sum of fat depots, all measured serum parameters and all measured parameters of the behavioral tests. Data from 60 animals were included in the analysis (10/group). Table 2 shows the variables with at least one significant correlation.

**Table 2:** Spearman correlation between body/serum parameters and behavioral variables.

	Swimming frequency	Immobility frequency	Latency to immobility	Climbing
Body weight gain(g)	−0.5233*	0.5514*	−0.3986*	−0.4669*
Feed efficiency (g/kcal)	−0.5467*	0.5765*	−0.3808*	−0.4856*
Sum of fat depots(g)	−0.3680*	0.3739*	−0.3732*	−0.3012*
Uterus (g)	0.5094*	−0.5699*	0.3668*	0.5219*
Glucose (mg/dL)	−0.1734	0.1616	−0.1622	−0.1224
Insulin (ng/mL)	−0.2937*	0.2986*	−0.2650*	−0.2627*
Homa-IR	−0.2859*	0.2847*	−0.2585*	−0.2341
Leptin (ng/mL)	−0.4452*	0.4815*	−0.3691*	−0.4441*

n = 60; *p < 0.05 (two-tailed).

In the FST test, the swimming frequency was negatively correlated to body weight gain (p < 0.001), feed efficiency (p = 0.003), sum of fat depots (p = 0.003), insulin levels (p = 0.025), HOMA-IR (p = 0.031) and leptin levels (p < 0.001) and positively correlated with uterus weight (p < 0.001). The immobility frequency showed positive correlation to body weight gain (p < 0.001), feed efficiency (p = 0.001), sum of fat depots (p = 0.001), insulin levels (p = 0.025), HOMA-IR (p = 0.034) and leptin levels (p < 0.001) while it correlated negatively to uterus weight (p < 0.001).

The latency to immobility showed negative correlation to body weight gain (p = 0.001), feed efficiency (p = 0.017), sum of fat depots (p = 0.002), insulin levels (p = 0.047), HOMA-IR (p = 0.047) and leptin levels (p = 0.003), and positive correlation to uterus weight (p = 0.007).

The climbing behavior showed negative correlation to the body weight gain (p < 0.001), feed efficiency (p = 0.006), sum of fat depots (p = 0.006), insulin (p < 0.001) and leptin levels (p < 0.001), while showing positive correlation to the uterus weight (p < 0.001).

Multiple linear regression models were constructed, using the same animals presented in Table 2, in order to identify predictors for the variations of behavioral parameters. All the variables which presented significant correlations with these behavioral parameters were tested in the models (Table 3).

**Table 3:** Multiple linear regression for behavior dependent variables.

Behavior	Predictor	Beta coefficient	Standard error	P-value	Adjusted R^2^
Swimming frequency	Uterus (g) Body weight gain(g)	13.500 −0.059	5.333 0.021	0.01414* 0.00758**	0.324
Immobility frequency	Uterus (g) Body weight gain (g)	−20.048 0.072	6.282 0.025	0.00230** 0.00591**	0.388
Climbing	Uterus (g) Body weight gain (g)	6.121 −0.017	2.076 0.008	0.00462** 0.04704*	0.297

n = 60; *p < 0.05, **p < 0.01.

The uterus weight was shown to significantly influence the swimming frequency as a positive predictor, and body weight gain as a negative one, explaining 32.4% of the variations.

The frequency of immobility was negatively influenced by uterus weight and positively influenced by body weight gain, and the equation explained 38.8% of the variations.

Climbing was positively influenced by uterus weight and negatively influenced by body weight gain. The equation predicted 29.7% of the climbing variations.

## Discussion

Ovariectomy alone increased body weight gain significantly and caused a 79% (p > 0.05) increase in body adiposity. This may be partly attributed to the observed increased feed efficiency, in agreement with our previous observations^12,25^, since the cumulative caloric intake of the OvxC group showed only a non-significant 12% increase in relation to that of the ShamC rats. Ovariectomy has been shown to stimulate food intake in rats^26^, although some studies have been shown that this effect is a transient one, disappearing after the first weeks after ovaries removal^27,28^.

Ovariectomy induced hyperleptinemia and hyperinsulinemia, indicating resistance to these hormones, the latter confirmed by increased HOMA-IR and HOMA-β values. High leptin levels have been described both in ovariectomized rodents and in post-menopausal women, and suggested to depend on the estrogen deficiency^8–10^. Our data are also in agreement with reports of hyperinsulinemia and insulin resistance in postmenopausal women^29^.

Estradiol replacement for twelve weeks failed to significantly restore body weight gain and feed efficiency in the control-fed rats. Actually, estradiol was effective in reducing these parameters significantly only up to week five. This could be attributed to a low subcutaneous estradiol replacement dose used in the present study, of 2.8 µg/day/90 days, in comparison to that (4.2 µg/day/60 days) reportedly able to decrease body weight gain and food intake and to increase metabolic rate of ovariectomized rats^30^. High doses of hormone replacement have also been shown to decrease body weight but not body adiposity in rats^31^.

In contrast, we presently observed that, by the end of the treatment, the hormonal replacement was sufficient to improve insulin and leptin levels, leptin/adiponectin ratio, and HOMA-IR and HOMA-β values. This agrees with the demonstrations that the anti-inflammatory properties of estrogen are related to its metabolic actions^32^ and that the chronic administration of an agonist of the estrogen receptor alpha improved the insulin sensitivity of diabetic mice^33^.

The intake of the high-fat diet by the non-ovariectomized rats (ShamL group) increased caloric intake and body adiposity but not body weight or feed efficiency, in comparison to the ShamC group. The effect of high-fat intake was more pronounced in the ovariectomized group, as it accentuated feed efficiency, body mass and adiposity gain, in relation to ovariectomy alone. This corroborates a study showing that ovariectomized mice were more vulnerable to weight gain and fat mass increase when fed a high-fat diet, due to high degrees of adipose tissue inflammation and pro-inflammatory citokynes production^34^.

The high-fat diet alone only tended to increase leptin and insulin levels while it did induce hyperglycemia, but no significant increase of HOMA-IR. Altogether, the results indicated that the consumption of this 45%-lard diet for 12 weeks had mild effects, perhaps due to the caloric and lipidic density of the diet as well as to the period of diet exposure. In agreement with this suggestion, other authors reported that the intake of a 45% lard-rich diet for 8 weeks by female rats failed to increase body fat percentage^35^, while a 62%-lard diet for 50 weeks doubled the body weight gain of female rats^36^. In male mice, the intake of a 60%-lard diet 16 weeks induced pronounced changes in body adiposity, glucose homeostasis, and leptinemia^37^.

The combination of ovariectomy and high-fat diet increased leptinemia and insulinemia, and led to significantly increased HOMA-IR and HOMA-β values, showing a further deterioration of the parameters affected by either ovariectomy or high-fat intake alone. These findings are consistent with the demonstration that impaired glucose homeostasis influence adipose tissue inflammation during high-fat intake^38^. The prominent increases in leptinemia induced by ovariectomy alone or combined with high-fat intake are likely to coexist with leptin resistance, probably due to multiple mechanisms including down–regulation and/or desensitization of hypothalamic leptin receptors and impairment of receptor signaling and leptin transport into brain^39^.

Differently from the observations in the control-fed rats, in the lard-fed ones the estradiol replacement failed to improve insulin and leptin levels, leptin/adiponectin ratio, and HOMA-IR, although it did reduce the HOMA-β value. It is hypothesized that, due to the poorer metabolic status of the lard diet-fed ovariectomized rats, the present estradiol treatment, of 2.8 µg/day, was not sufficient. This suggestion agrees with a similar experiment in ovariectomized mice fed a high-lard diet, in which a dose of 1.7 µg/day, high in comparison to the present one in rats, protected from insulin resistance^9^^.^

Having demonstrated the high-fat intake potentiated the alterations induced by ovariectomy, we decided to evaluate behavioral aspects in these animals, due to the controversy around the incidence of mood disorders and its relation to obesity after menopause^5,22^.

In the ovariectomized animals fed with the control diet, we found no significant effects on the depressive-like behaviors measured in the FST. While these results agree with previous reports in rodents utilizing the same behavioral paradigm^12,19–21^, other studies have found ovariectomy to induce depressive-like behaviors^11,17,18^. These contradictory fiings may be attributed to methodological discrepancies among these studies. Indeed, depressive behaviors have been observed when either ovariectomy was performed at an earlier age or the behavioral test was performed more acutely after the surgery, agreeing with review papers in humans concluding that the risk to develop depression was inversely associated with the age at menopausal onset^2^ and that the menopausal transition is a period of vulnerability to the development of depression^22^.

The high-fat diet alone also failed to induce depressive-like behaviors in the FST, a result apparently inconsistent with the epidemiological data linking obesity and depression^5^. This may be attributed to the relatively mild degree of the alterations induced by the present high-fat regimen, as discussed above. Accordingly, a more potent obesogenic regimen in male mice induced depressive-like behaviors, which was attributed to both high-fat intake and impaired central leptin signaling^37^.

In contrast, the ovariectomized group fed with the high-lard diet exhibited clear depressive-like behaviors in the FST. Importantly, no deficit in locomotor activity was observed during the EPM test, ruling out that impaired mobility due to obesity influenced the results, as previously suggested^40^.

We have previously demonstrated that the combination of ovariectomy and high-lard intake decreased the latency to immobility in the FST but, unlike the present results, failed to affect the immobility time and the swimming frequency, indicating lack of a consistent depressive effect^12^. This difference may be related to the present longer period of diet treatment after ovariectomy (12 weeks), leading to potent accentuation of body adiposity and metabolic impairment, in comparison to our previous study (8 weeks). These findings agree with epidemiological studies linking visceral fat and depressive symptoms in middle-aged women and appointing visceral adiposity as the factor responsible for increased risk of depression in postmenopausal women^13,14^. Moreover, these findings corroborate the present correlation and regression data showing that the depressive-like behaviors were significantly influenced by the metabolic and body composition parameters and by the estrogen status, as indicated by uterus weight.

The present estradiol replacement regimen failed to consistently reverse the depressive-like behavior observed in the OvxL group, probably due to a low dosage, as discussed above. This suggestion agrees with the notion that estrus levels of estradiol are necessary to decrease depression behavior in rats^16^.

In conclusion, the present data indicate that the vulnerability to develop depression after menopause is positively influenced by obesity. It is suggested that weight management is a crucial issue in postmenopausal women, probably having a beneficial role in preventing the appearance of mental health problems.

## Material and Methods

### Animals and surgery conditions

All procedures were approved by and conducted according to the guidelines of the Committee in Research Ethics of the Universidade Federal de São Paulo (CEUA No.: 2172030315/ 2016), following the ethics procedures stablished by the Conselho Nacional de Controle de Experimentação Animal (CONCEA). Twelve-week-old female Wistar rats were submitted to either bilateral ovariectomy (Ovx, n = 71) or sham operation (Sham, n = 35) under ketamine/xylazine anesthesia (66/33 mg/kg, ip). For estrogen replacement, 35 Ovx animals were implanted with a subcutaneous pellet of 17β-estradiol (0.25 mg/pellet, 90-day release; Innovative Research of America, Sarasota, Florida, USA). Immediately after the surgery, all animals received penicillin (60.000U, i.m.) and ibuprofen (1 mg/kg body weight, v.o.).

Additional ibuprofen doses were administered once daily for 2 days. After the surgery all the animals were housed two to three per cage and maintained under controlled lighting (12 h light/dark cycle, lights on at 6am) and temperature conditions (23 ± 1 °C), with free access to food and water. Upon housing, the above groups were randomly sub-divided according to the diet offered. ShamC, OvxC and OvxC + E2 received standard rat chow (2.87 kcal/g, 15% of energy from fat, Nuvilab CR-1, Nuvital Nutrientes SA, Colombo, PR, Brazil) while the ShamL, OvxL and OvxL + E2 groups received a high-fat diet (3.60 kcal/g, 45% energy from fat) enriched with lard (Cooperativa Central Aurora de Alimentos, Chapecó, Santa Catarina, Brazil) to the chow diet. The high-fat diet was prepared by adding, to the standard chow, 18% (w/w) lard, 2% soybean oil, 20% (w/w) casein, 10% sucrose and 0.02% (w/w) of butylhydroxytoluol. Body weight and feed efficiency were measured weekly. Food and caloric intake were measured as the mean consumption value for each housed rat, during the treatment. Feed efficiency was calculated as follows: (body weight gain/energy intake) × 100^36^.

### Behavioral assessment

#### Elevated plus-maze (EPM)

The test apparatus consists of two open arms and two closed arms connected by a central platform high off the ground and lit by a dim light. On the day of the test, the rats were transported individually into the testing room and placed on the central platform facing an open arm. The test was performed and recorded during five minutes by an overhead video camera for later quantification. The number of entries in the arms was measured by two observers^41^. The number of entries in the enclosed arms and the total number of entries evaluate locomotor activity^42–45^.

#### Modified forced swim (FST)

At the first day of the tenth week, the rats were transported to the FST test room and placed individually in a plexiglas cylinder filled with water (diameter 30 cm, height 50 cm) for the 15-minutes training session. The volume of water (25 ± 1 °C) was enough to prevent the animal from touching the bottom with the tail. After 24 hours, the 5-minutes test session was performed while being recorded for subsequent analyses. Every 5 seconds, the predominant behavior was characterized as swimming (movements throughout the swim cylinder), climbing (upward-directed movements with the forepaws along the cylinder walls) and immobility (floating with minimal movements with head just above the water). Latency (total of seconds before the first immobility account) was also assessed^46^.

### Serum measurements

At the end of the twelfth week, the rats were euthanized after 24 hour fast (after thiopental anesthesia), trunk blood was collected and serum stored at −80 °C until analyzed. Uterus, liver, retroperitoneal, gonadal and mesenteric fat pads were dissected and weighed.

Serum glucose, cholesterol, HDL-cholesterol and triglycerides levels were determined using commercially available enzymatic colorimetric kits (Labtest Diagnóstica, Lagoa Santa, MG, Brazil). Elisa kits (Millipore, Bedford, MA, USA) were used to determine the serum levels of leptin (sensitivity 0.08 ng/mL; intra assay precision- 2.49%; inter-assay precision- 3.93%), insulin (sensitivity 0.1 ng/mL; intra-assay precision- 1.33%; inter-assay precision- 6.71%) and adiponectin (sensitivity – 0.4 ng/mL; intra-assay precision- 1.18%; inter-assay precision- 7.34%). The leptin/adiponectin ratio was then calculated. The Homeostasis Model Assessment Insulin Resistance index (HOMA-IR) was calculated as follows: HOMA-IR = (fasting insulin (µM/mL) x fasting glucose (mmol/L))/22.5). HOMA-ß was calculated as follows: HOMA-ß = (insulin x20)/(glucose-3.5)^47^.

### Data analysis

Statistical analysis was performed in Statistica 12 Software (StatSoft, Tulsa, OK, USA). The variables were tested for distribution and homogeneity (Shapiro-Wilks and Levene test respectively). Parametric variables were expressed as means ± standard error, and analyzed by two-way analysis of variance (ANOVA) with Tukey post hoc test. The non-parametric variables were expressed as median (interquartile range) and analyzed by Kruskal-Wallis ANOVA followed by two tailed multiple comparisons of p-values.

The interaction between variables was tested by the Spearman´s correlation coefficient. Multiple linear regressions were applied to determine the predictors of behavioral parameters among the variables presenting significant correlations. The level for statistical significance was set at p < 0.05.
